# Supplementary material for: The receiver operating characteristic curve accurately assesses imbalanced datasets
Source: Patterns (N Y). 2024 May 31;5(6):100994. doi: 10.1016/j.patter.2024.100994 (PMC11240176; doi:10.1016/j.patter.2024.100994)
Supplement: Document S1. Figures S1–S5, Table S1, and Proof S1 [file mmc1.pdf]

Patterns, Volume *5*

Supplemental information

The receiver operating characteristic curve accurately assesses imbalanced datasets

Eve Richardson, Raphael Trevizani, Jason A. Greenbaum, Hannah Carter, Morten Nielsen, and Bjoern Peters

Supplemental Experimental Procedures

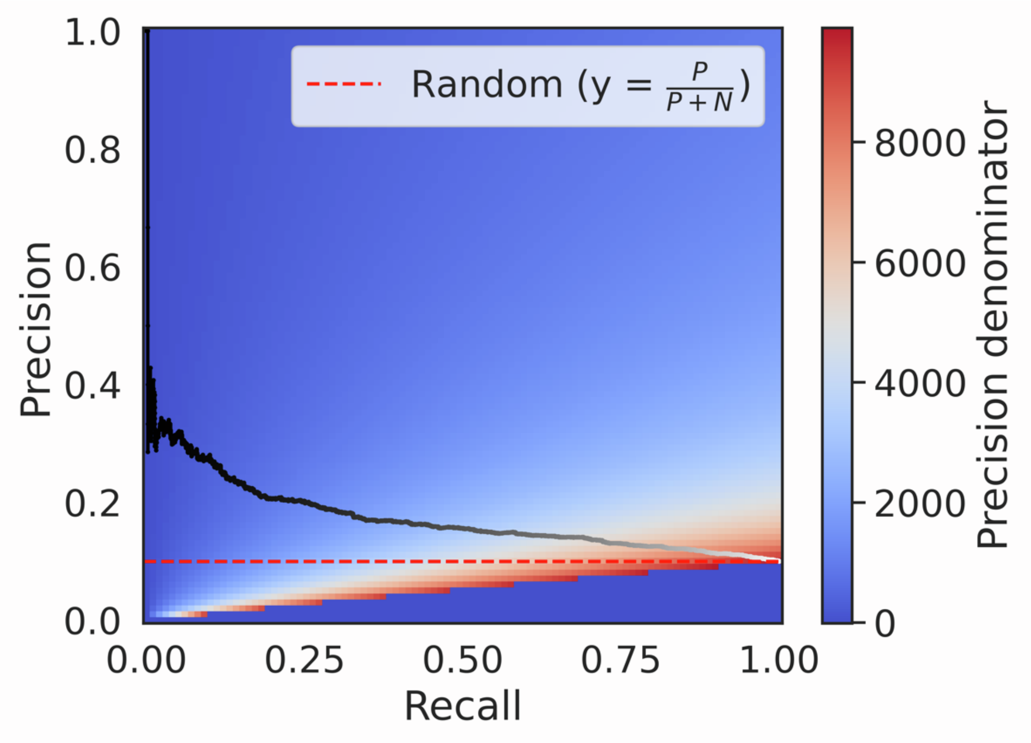

Figure S1: PR space is non-linear, as the denominator of Precision ($\hat{P}$) changes while moving through PR space which means that Precision does not vary uniformly with TPR. At the lowest score threshold, $\hat{P}$ will equal P+N (i.e. everything predicted as positive), and will decrease from right to left across the plot (until nothing is predicted as positive). The dark area below the random baseline corresponds to the unachievable area for a given TPR level, defined by the imbalance in the dataset [1]. With an imbalance of 0.1, Precision is only defined at TPR levels greater than 0.053 according to the formula presented in Boyd et al (2012). This limits the extent to which classifiers can be worse than random.

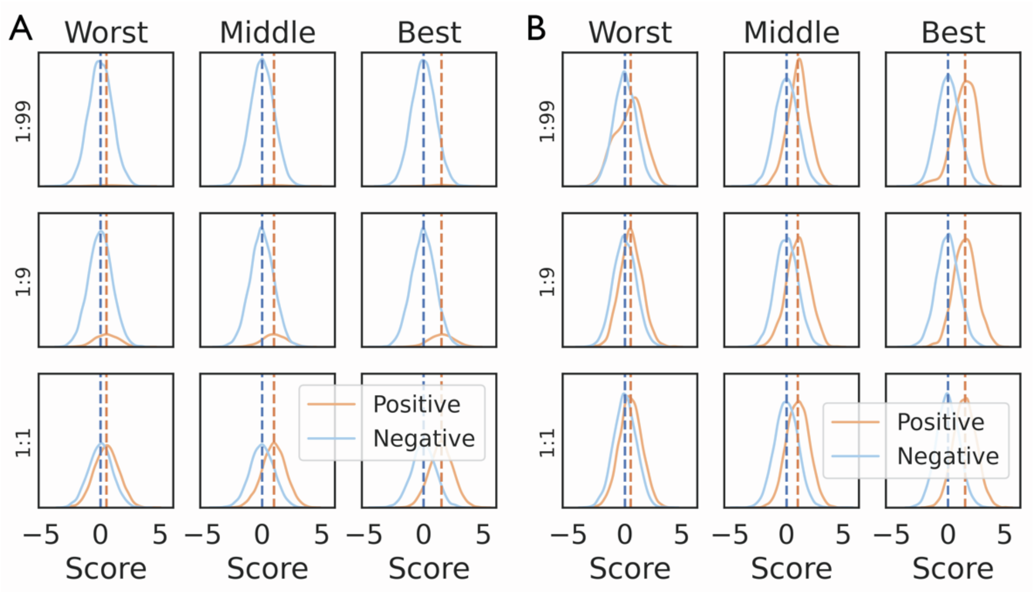

Figure S2: total score distributions across all simulations for each class imbalance (rows) and each classifier (columns), normalized across the entire dataset (A) and on a per-class basis (B). The underlying score distributions for the positive and negative class is not changed by changing class imbalance.

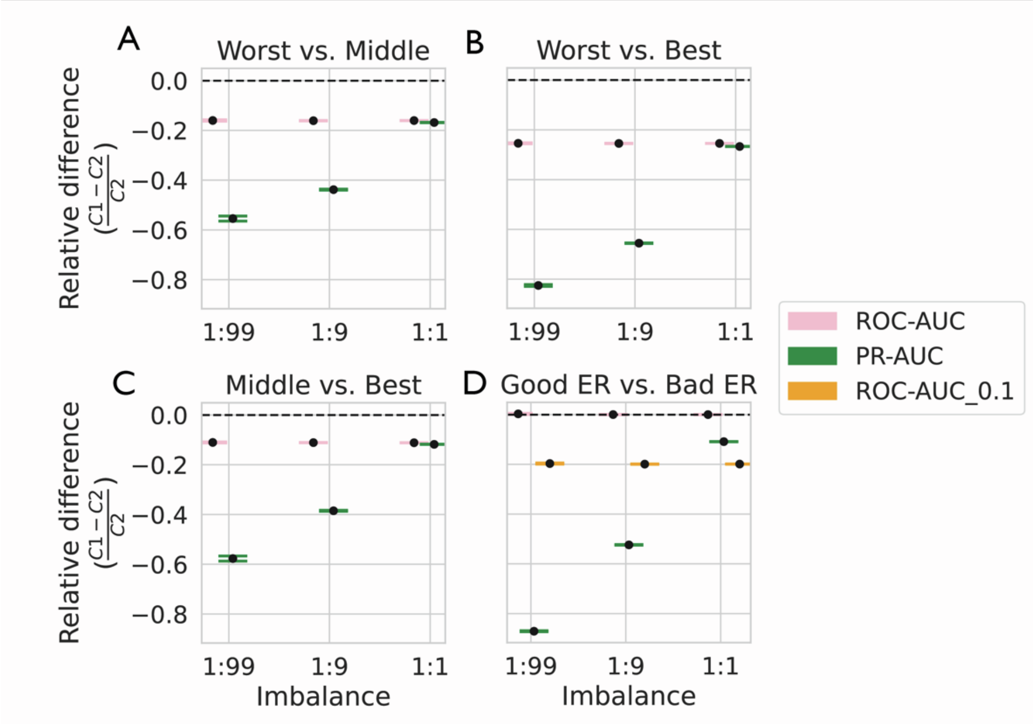

Figure S3: classifier performance differences between each pair of classifiers, “Worst”, “Middle”, “Best” (A, B, C) and the Good vs. Bad ER (D). The metric for the better classifier (C2) is subtracted from the worse classifier (C1) and divided by the performance estimate of the better classifier to calculate the relative difference, (C1 -

C2)/C2, for each imbalance. ROC-AUC's estimate of the difference in performance is constant as expected given that the absolute performance estimation is constant. Meanwhile, the difference in the performance per the PR-AUC differs by as much as 80% of the value of the better classifier. While the ROC-AUC (pink) is not able to distinguish between classifiers with good and bad early retrieval and symmetrical distributions because the corresponding curves cross (D), a partial AUC over the early retrieval region such as ROC-AUC_0.1 (orange), allows us to distinguish between the two (i.e. non-zero difference), irrespective of the underlying class distribution. The PR-AUC (green) is sensitive to early retrieval behavior due to the fact that it explicitly only considers the positive class, however it is also highly sensitive to the underlying class imbalance.

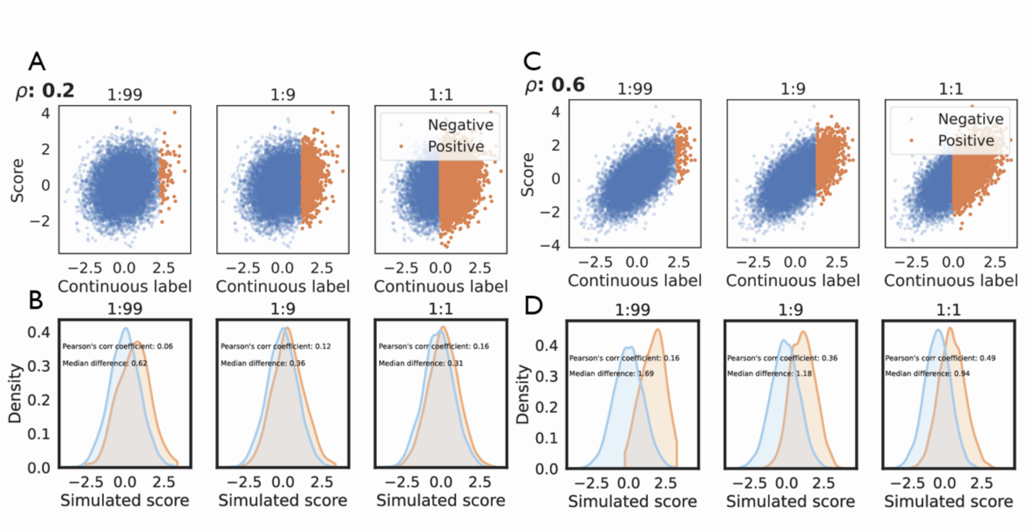

Figure S4: we recreated the simulation framework from Cook & Ramadas (2020), in which classifier performance is simulated via the covariance between two zero-centred Normal distributions (implemented with numpy's multivariate_normal function)[2]. Shown here are a covariance of 0.2 (A and B) and 0.6 (C and D). In this simulation, we convert the continuous label into a binary label via selecting a threshold above which the top 1%, 10% or 50% of instances are labelled as positive (A, C). This results in an improvement in the actual simulated classifier performance with increasing imbalance where the correlation coefficient between the two distributions is non-zero, as then the top 1%, 10% or 50% of instances by the label distribution is also likely to be among the top scoring instances in the score distribution (B and D).

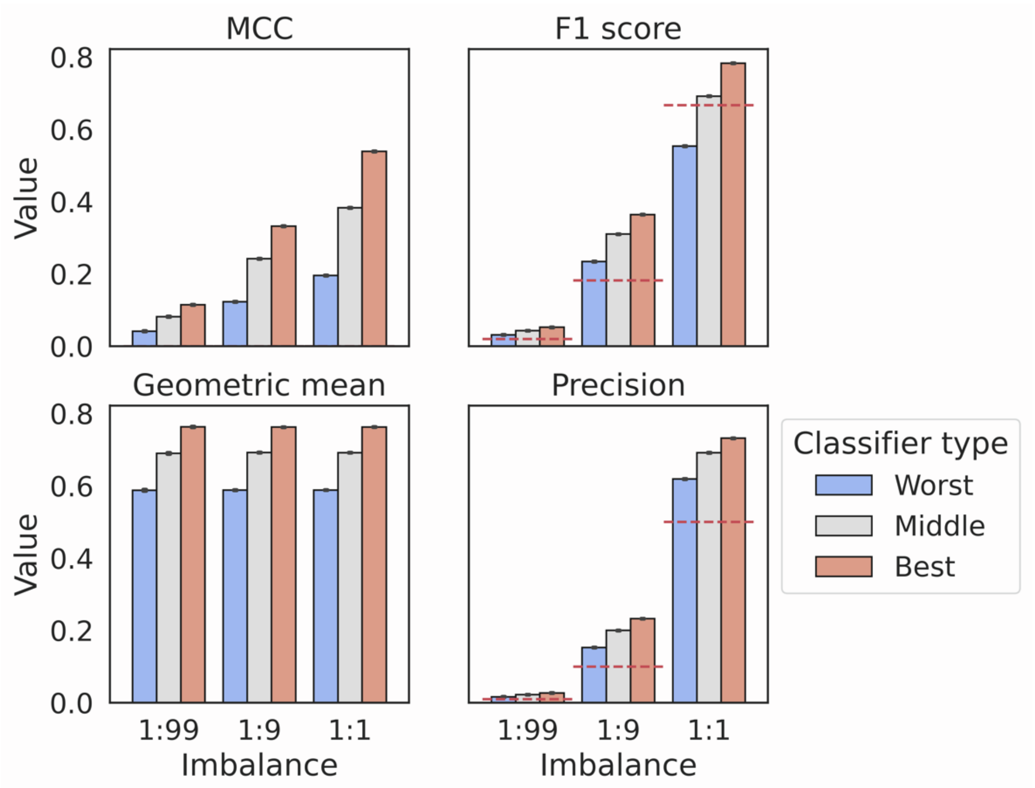

Figure S5: we calculated single-threshold performance estimates for our simulated classifiers, using an arbitrary score threshold of 0.5 for each classifier. Red dashed lines indicate the random baseline where all instances are predicted as positive, i.e. where Precision = Imbalance and Recall = 1. For MCC and Geometric Mean, the random baseline is 0. For the PR metrics (MCC and F1-score), classifier performance and deviation from random baseline is a function of imbalance. The geometric mean is constant across different imbalances (geometric mean of TPR and (1-FPR)).

	Imbalance		
Correlation	1:99	1:9	1:1
0.0	0.50 / 0.50	0.50 / 0.50	0.50 / 0.50
0.2	0.66 / 0.65	0.61 / 0.61	0.59 / 0.59
0.4	0.79 / 0.79	0.72 / 0.72	0.68 / 0.68
0.6	0.90 / 0.90	0.82 / 0.82	0.78 / 0.78
0.8	0.97 / 0.97	0.92 / 0.92	0.88 / 0.88

Table S1: median ROC-AUC across our 1,000 simulations vs. reported ROC-AUC by Cook & Ramadas (2020), with varying classifier performance (correlation between the two distributions which Cook & Ramadas denote as ρ) and imbalance. Note that the

ROC-AUC looks to be “inflated” with increasing class imbalance: this is just the ROC-AUC correctly reflecting the fact that the simulated classifier performance is indeed better in more imbalanced datasets in this simulation framework, due to the imbalance changing the score distribution. This effect is more pronounced where the two distributions are more correlated, e.g., the difference from a balanced to the least balanced simulation is 0.7 with a covariance of 0.2, and 0.12 with a covariance of 0.8.

Supplementary Proof 1:

$$TPR = \frac{TP}{P}$$

$$FPR = \frac{FP}{N}$$

$$PPV = \frac{TP}{TP + FP}$$

$$PPV = \frac{P \cdot TPR}{P \cdot TPR + N \cdot FPR}$$

$$PPV = \frac{TPR}{TPR + \frac{N}{P} \cdot FPR}$$

1. Boyd, K., Santos Costa, V., Davis, J., and Page, C.D. (2012). Unachievable Region in Precision-Recall Space and Its Effect on Empirical Evaluation. Proc. Int. Conf. Mach. Learn. Int. Conf. Mach. Learn. *2012*, 349.

2. Cook, J., and Ramadas, V. (2020). When to consult precision-recall curves. Stata J. *20*, 131–148. 10.1177/1536867X20909693.
