# Supplementary material for: The receiver operating characteristic curve accurately assesses imbalanced datasets
Source: Patterns (N Y). 2024 May 31;5(6):100994. doi: 10.1016/j.patter.2024.100994 (PMC11240176; doi:10.1016/j.patter.2024.100994)
Supplement: Document S2. Article plus supplemental information [file mmc2.pdf]

Article

Patterns

The receiver operating characteristic curve accurately assesses imbalanced datasets

Graphical abstract

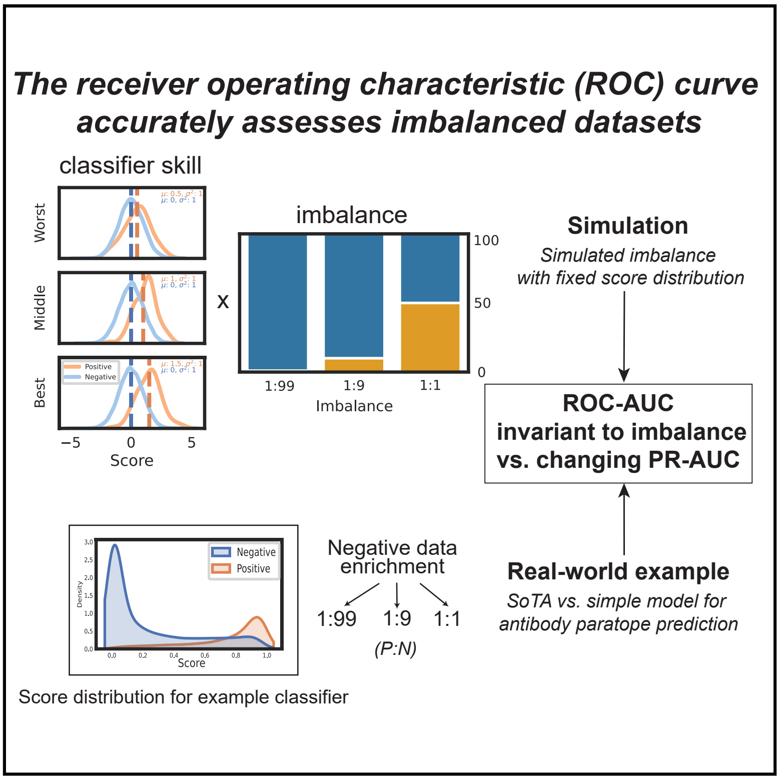

Highlights

- For imbalanced datasets, recent papers report that the ROC-AUC is inflated
- Simulated and real-world data show that the ROC-AUC is invariant to class imbalance
- The effect of class imbalance on the PR-AUC cannot be trivially removed
- Partial ROC-AUCs allow performance evaluation over the upper score ranges

Authors

Eve Richardson, Raphael Trevizani, Jason A. Greenbaum, Hannah Carter, Morten Nielsen, Bjoern Peters

Correspondence

bpeters@lji.org

In brief

In datasets where there are many more negative than positive instances, it has become common wisdom that the ROC-AUC is inflated and the PR-AUC should be used. The authors show that this is a misunderstanding: the ROC-AUC is invariant to class imbalance when the score distribution isn't changed by the imbalance, in contrast to the PR-AUC. The ROC-AUC and its early retrieval area allow an estimate of classifier performance vs. PR-AUC's estimate of classifier performance on a particular dataset.

Check for updates

Richardson et al., 2024, Patterns *5*, 100994
June 14, 2024 © 2024 The Authors. Published by Elsevier Inc.
https://doi.org/10.1016/j.patter.2024.100994

CellPress

Patterns

CellPress
OPEN ACCESS

Article

The receiver operating characteristic curve accurately assesses imbalanced datasets

Eve Richardson,[1] Raphael Trevizani,[1,2] Jason A. Greenbaum,[1] Hannah Carter,[3] Morten Nielsen,[4] and Bjoern Peters[1,5,*]
[1]Center for Infectious Disease and Vaccine Research, La Jolla Institute for Immunology, La Jolla, CA, USA
[2]Fiocruz Ceará, Fundação Oswaldo Cruz, Rua São José s/n, Precabura, Eusébio/CE, Brazil
[3]Department of Medicine, University of California, La Jolla, CA, USA
[4]Department of Health Technology, Section for Bioinformatics, Technical University of Denmark, Lyngby, Denmark
[5]Lead contact
*Correspondence: bpeters@lji.org
https://doi.org/10.1016/j.patter.2024.100994

THE BIGGER PICTURE There is conflicting information about whether a popular metric, the area under the curve (AUC) of the receiver operating characteristic (ROC) curve, should be used when a dataset has many more negatives than positives, and one is only interested in performance on the positive instances. Many practitioners prefer a metric called the precision-recall (PR)-AUC and are taught that the ROC-AUC will give an "overly optimistic" estimate of model performance. We show that the ROC-AUC is only inflated by an imbalance in simulations where changing the imbalance changes the score distribution. By contrast, the PR-AUC changes drastically with class imbalance; furthermore, one cannot subtract or normalize the PR-AUC by class imbalance to obtain a corrected performance estimate. Our work encourages the adoption of the ROC-AUC in such cases, allowing for fairer comparisons of models across datasets with different imbalances and furthering the understanding of the relationship between the ROC and PR spaces.

SUMMARY

Many problems in biology require looking for a "needle in a haystack," corresponding to a binary classification where there are a few positives within a much larger set of negatives, which is referred to as a class imbalance. The receiver operating characteristic (ROC) curve and the associated area under the curve (AUC) have been reported as ill-suited to evaluate prediction performance on imbalanced problems where there is more interest in performance on the positive minority class, while the precision-recall (PR) curve is preferable. We show via simulation and a real case study that this is a misinterpretation of the difference between the ROC and PR spaces, showing that the ROC curve is robust to class imbalance, while the PR curve is highly sensitive to class imbalance. Furthermore, we show that class imbalance cannot be easily disentangled from classifier performance measured via PR-AUC.

INTRODUCTION

Binary classification, and specifically binary classification where positive instances are greatly outnumbered by negative instances, frequently occurs in biological problems. For example, protein-protein and protein-ligand interactions,[1,2] oncogenic mutations,[3] species occurrence,[4] diagnostic testing,[5] and antibody and T cell receptor epitope prediction[6,7] are domains in which the positive instances are outnumbered greatly by negative instances. These are examples of problems with class imbalance: class imbalance describes an unequal distribution of instances among classes "understood [within the community] to correspond to datasets exhibiting significant and, in some cases, extreme imbalance."[8] Extreme imbalance corresponds to orders of magnitude differences in class distribution. The ability to accurately and reproducibly describe and rank classifier performance in the context of such imbalanced data is therefore critical to multiple important tasks in biology.

All binary classification metrics are calculated using the confusion matrix, a contingency table that specifies the relationship between the ground truth and the labels predicted by a classifier at a particular operating point, i.e., a particular threshold score above which instances are predicted as positive (Figure 1A). Different quadrants of this confusion matrix are used to calculate measures such as accuracy, precision, false positive rate (FPR) and the true positive rate (TPR), or recall, which are used to assess model performance (Figure 1B). When an optimal operating point is not known beforehand, curves that consider

This is an open access article under the CC BY license (http://creativecommons.org/licenses/by/4.0/).

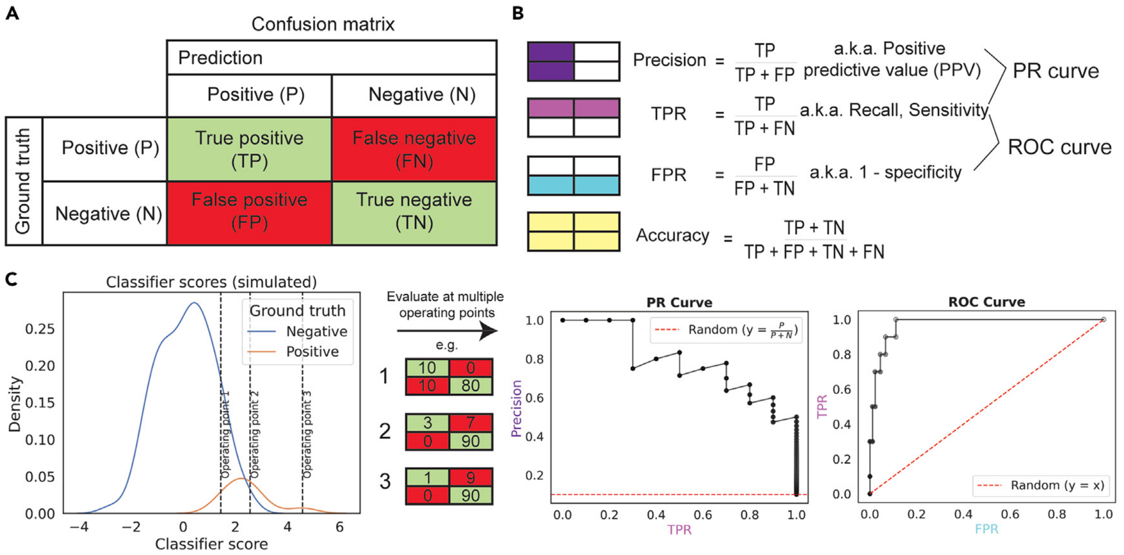

Figure 1. The ROC and PR curves are calculated from different quadrants of the confusion matrix over multiple operating points

(A) For binary classification, a confusion matrix can be calculated for the classifier at a particular operating point (e.g., a score threshold, as shown here).

(B) From the confusion matrix, metrics such as precision, TPR (otherwise known as recall or sensitivity) and FPR (which is equal to 1 − specificity) can be calculated. Precision and TPR define the PR space, while TPR and FPR define the ROC space.

(C) To produce a PR or ROC curve, these metrics are calculated at multiple operating points. These points can be interpolated, and the areas under the resulting curve can be calculated and compared across classifiers.

performance over a range of operating points are useful (Figure 1C). Curve inspection allows the assessment of how performance changes as a function of threshold, and the area under the curve (AUC) provides a summary metric to compare classification performance across curves. These terms are defined in Table 1.

Accuracy is an inappropriate measure to use for imbalanced datasets where there is interest in predictive performance on the minority class because then a score equal to the class imbalance (which is by definition high in imbalanced datasets) can be achieved by predicting every instance as the majority class.[9] The receiver operating characteristic (ROC) curve, which was invented in the context of signal detection theory and widely used in diagnostics, addresses this problem by its use of complementary pairs within the confusion matrix, expressed proportionally to the number of positive and negative instances and therefore independent of the underlying class imbalance.[10] The ROC curve's axes use combinations of all quadrants of the confusion matrix: the FPR, the proportion of the total negative dataset falsely predicted as positive, on the x axis and the TPR, the proportion of the total positive dataset correctly predicted as positive, on the y axis (Figure 1). Despite the popularity of the ROC curve in many fields, many practitioners find it more intuitive to understand their model performance with the precision-recall (PR) curve, which uses precision (rather than the FPR) in addition to TPR (which is referred to as recall in the PR curve case).[11]

As described by Flach and Kull,[12] the ROC space has a number of convenient properties that the PR space lacks. Firstly, the ROC curve's invariance to class imbalance results in a universal random baseline AUC of 0.5, whereas the random baseline AUC for the PR curve is equal to the imbalance of the dataset. The ROC-AUC has numerous interpretations such as its relatedness to the Mann-Whitney U statistic, the Wilcoxon signed-rank test, and the Brier score,[13,14] and due to the linear nature of the ROC space, its calculation via the trapezoidal or midpoint method is unambiguous. By contrast, the PR-AUC lacks an interpretation beyond an average precision weighted by different recall values.[12] Furthermore, PR space is non-linear because of the changing denominator of the positive predictive value (PPV) (Figure S1).

Despite ROC curves being established within the ROC literature to be invariant to class imbalance, PR has, interestingly, in recent years become widely recommended as preferable to the ROC space in the case of severely imbalanced datasets. This appears to originate with an aside in Davis and Goadrich's well-cited work[15] establishing the correspondence between the PR and ROC space, in which they argue that "ROC curves can present an overly optimistic view of an algorithm's performance if there is a large skew in the class distribution." This is not shown empirically or numerically, with the only justification being the intuition that "a large change in the number of FPs can lead to a small change in the FPR used in ROC analysis."[15]

This paper has since been widely cited including in textbooks and reviews in imbalanced learning, as well as in the documentation for the corresponding functions in the popular machine learning Python package scikit-learn.[8,16–20] Subsequently, there have been a number of studies using simulations or comparisons on domain-specific datasets[4,11,21–24] that have supported the claim that ROC-AUC inflates performance estimates of classifiers on imbalanced datasets. That PR-AUC is preferable to the ROC-AUC for imbalanced datasets has become "common knowledge" in many subfields, for example, in antibody paratope and epitope

CellPress
OPEN ACCESS

Table 1. Definition of terms used in the article

Term	Definition
Ground truth	the "known" label for an instance in a dataset; in binary classification, this is a 1 or 0, which are here referred to as positives (P) and negatives (N), respectively
Confusion matrix	a 2 × 2 contingency table in which the rows are the ground-truth labels (P or N) and the columns are the predicted labels ($\hat{P}$ or $\check{N}$), or vice versa; the quadrants are true positives (TPs) and true negatives (TNs), which are positive and negative instances successfully classified as such, and false positives (FPs) and false negatives (FNs), which are negatives falsely classified as positives and positives falsely classified as negatives respectively
Operating point	an operating point is a specific use of a classifier output or set of classifier outputs; most commonly, this refers to the selection of a score threshold where the classifier has a continuous (not necessarily calibrated) output, with instances scoring above this threshold being labeled as positives and instances scoring below labeled as negatives
Precision; positive predictive value	TP/(TP + FP) = TP/$\hat{P}$
True positive rate; recall; sensitivity	TP/(TP + FN) = TP/P
False positive rate; 1 − specificity	FP/(FP + TN) = FP/N
Specificity	TN/(FP + TN) = TN/N
Accuracy	(TP + TN)/(TP + FP + FN + TN) = (TP + TN)/(N + P)
ROC curve	a plot in which the x axis is FPR and the y axis is TPR; each point corresponds to a particular operating point, e.g., score threshold; straight lines are drawn between each point
PR curve	a plot in which the x axis is TPR (referred to as recall) and the y axis is precision; as in the ROC curve, each point corresponds to a particular operating point, and lines are drawn between each point to construct a curve
AUC	area under the curve, e.g., ROC (ROC-AUC) or PR (PR-AUC) curve, providing a single number that summarizes predictive performance, allowing comparison of curves
Early retrieval (ER)	performance of the classifier between up to a certain FPR range, e.g., FPR 0 to FPR_{max} = 0.1; this reflects the performance of the classifier over the highest-scoring instances

prediction, for which recent papers have favored PR over the ROC-AUC for this reason.[25–28]

The clear disagreement between the ROC literature (in which the ROC curve is shown to be robust to class imbalance), and the claims of Davis and Goadrich and many subsequent researchers has not been examined critically and has contributed to widespread confusion about the ROC curve's place in evaluating classifiers on imbalanced datasets. We argue that the common recommendation of the use of the PR rather than ROC space for imbalanced datasets is the result of a misunderstanding about the relationship between the ROC and PR curves, with the PR curve's use of precision reflecting classifier performance on a specific dataset with its specific imbalance but with limited generalizable value. We show via simulation that ROC-AUC, for a given classifier skill, is robust across multiple datasets with different class imbalances, in contrast to PR-AUC. We emphasize that the ranking of classifiers with different predictive performance by ROC-AUC across different class imbalances is stable, demonstrating that the ROC-AUC does not lose or downweigh information about positive predictive performance with increasing class imbalance. We then demonstrate this in a real-world example, antibody paratope prediction. Furthermore, we show that the class imbalance that affects the construction of the PR curve cannot be trivially subtracted or normalized from the resulting PR-AUC. Finally, we demonstrate how performance assessment can be easily limited to early retrieval (ER) via the partial ROC-AUC if practitioners prefer to focus on their model's performance on the positive class.

RESULTS

The ROC-AUC provides a robust estimation and ranking of classifier performance across different class imbalances, while PR-AUC changes drastically

We simulated three classifiers with varying performances, which we refer to as "worst," "middle," and "best." The total dataset size was fixed at 10,000, and class imbalance was introduced by varying the ratio of positive instances (P) to negative instances (N) between 1:99, 1:9, and 1:1. In our selected simulation style, negative instances are drawn randomly from a defined score distribution, so the score distribution is not affected by class imbalance. We consider this the optimal simulation style because it allows us to disentangle imbalance from changes in the simulated score distribution.

The simulated score distributions across these classifiers for a random simulation at an imbalance of 1:99 with per-class normalization are shown in Figure 2A, and the resulting distributions of PR- and ROC-AUC across varying classifier performance and class imbalances are shown in Figure 3. Scores are

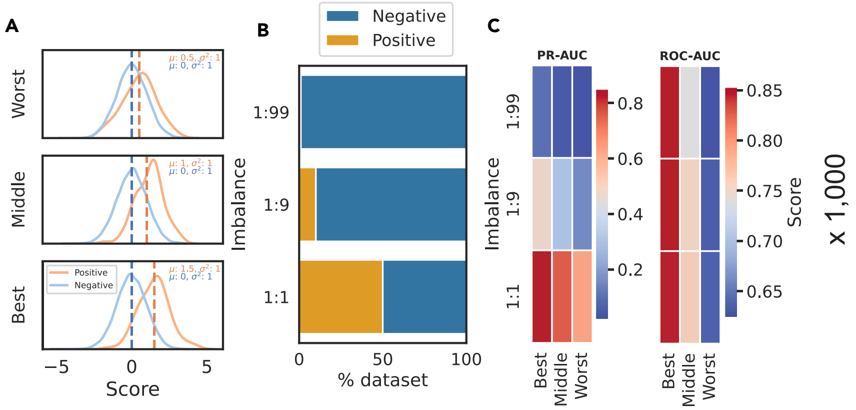

Figure 2. Our simulation framework simulates varying classifier performance under different imbalances

(A) We simulated three classifier performances defined by the mean centering of the simulated positive scores from "worst" with a positive score distribution centered at 0.5 to "best" with a positive score distribution centered at 1.5.

(B) We simulated a dataset size of 10,000 instances with three class imbalances where imbalance is defined as P:N.

(C) For each pair of imbalances and classifier types, we calculated PR- and ROC-AUCs. This was repeated 1,000 times.

shown with per-class and total class normalization across all imbalances in Figure S2.

In our simulations, the PR-AUC (and its random baseline, indicated as a red dashed line) changes dramatically with class imbalance for a classifier of a given skill level, while the ROC-AUC's median value (and its random baseline) is constant. In conclusion, the ROC-AUC of a classifier is not inflated by class imbalance of the dataset it is being evaluated on in any way; in fact, it is immutable to it. In contrast, the PR-AUC is highly sensitive to changes in class imbalances of the dataset it is evaluated on because it reflects precision at a particular class imbalance.

The ER area of the ROC curve is more informative about performance on the positive class

We have so far considered the area under the entire ROC curve equally. When there is more interest in performance on the positive class, the area of ROC space to focus on is referred to as the ER area, reflecting the area in which the FPR is lowest.[29] The fact that different areas of the ROC curve correspond to performance over different parts of the score distribution also means that it is not advised to directly compare AUCs if the underlying ROC curves cross (Fawcett[30]).

We simulated classifiers with different performance over the ER area at the same imbalances as in Figure 3 (Figures 4A–4C). The corresponding ROC and PR curves and AUCs are calculated as the area under the ER region of the ROC curve, here defined by an FPR_{max} of 0.1 (which corresponds to classifying 10% of negative instances as positives), with the AUC referred to as ROC-AUC_0.1 as in other work.[31–33]

The criticism by Saito and Rehmsmeier[11] was that the PR-AUC is able to distinguish between two classifiers with very different score distributions that result in differing ER behavior (Figure 4D), while ROC-AUC is not (Figure 4E). In this case, plotting the ROC curves reveals that the "good ER" classifier is superior in the ER area, while the "bad ER" classifier is superior in the late retrieval

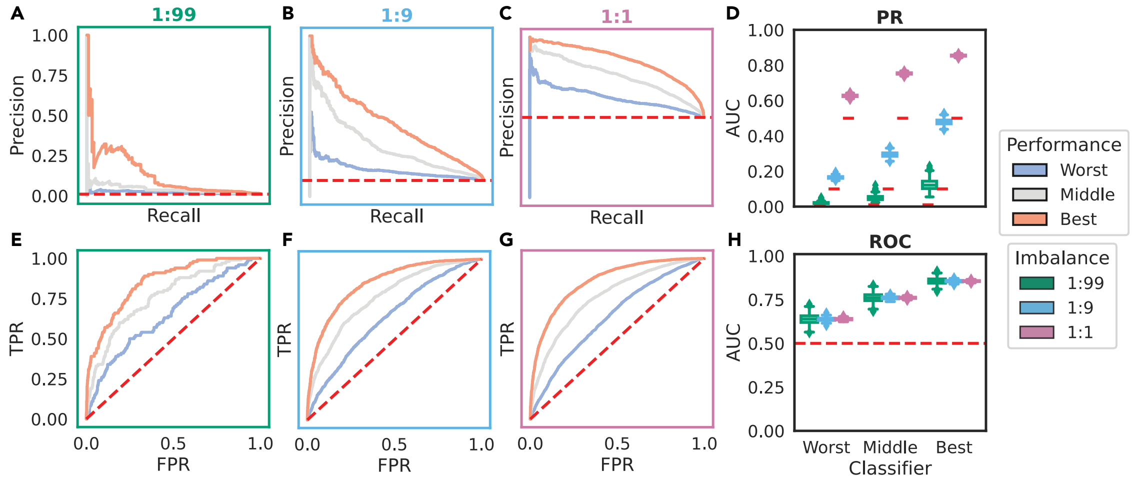

Figure 3. The PR curve and PR-AUC are highly sensitive to class imbalance, while the ROC curve and ROC-AUC are constant

(A–D) The PR curve for each classifier (worst in blue, middle in gray, and best in orange) changes with the underlying class imbalance (A–C), resulting in a changing AUC (D), despite a constant underlying score distribution (Figure S2).

(E–H) The ROC curve (E–G) and AUC (H), as well as its random baseline, stay constant with varying imbalance.

The curves shown are for a single representative simulation, while the distribution of the PR- and ROC-AUCs across 1,000 simulations at each class imbalance are shown in (D) and (H), respectively. PR-AUC changes drastically with the class imbalance, while the ROC-AUC distribution across simulations is constant. This is a fundamental property of the ROC curve.

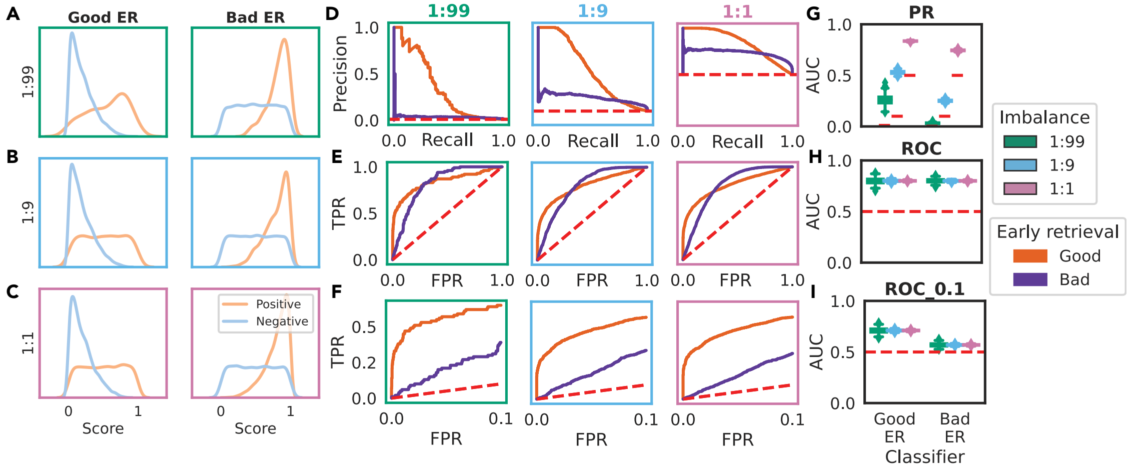

Figure 4. ROC-AUC_0.1 can distinguish between classifiers with different ER behavior

ROC curves and associated AUCs are calculated across the full threshold range of a classifier by default, which may not be optimal if specifically interested in predictive performance on the positive class. The AUC can instead be calculated over the early retrieval (ER) region, defined in terms of the FPR limit (FPR_{max}).

(A–C) We simulated classifiers with good and bad ER over a range of imbalances.

(D–I) For a classifier with good and bad ER behavior, both PR (D) and ROC (E) curves reveal performance differences in the classifier performance. However, while the PR-AUC reveals differing classifier performance in a class imbalance-dependent manner (D), AUC calculation over the full ROC curve cannot distinguish the two classifiers (E), which is why it is recommended not to compare AUCs where ROC curves cross. Simply calculating the AUC up to an FPR below the crossing points reveals the difference between these two classifiers: a partial AUC is calculated from the ROC curve and can be scaled via the McClish correction so that random performance is still equal to 0.5 (F). In contrast to the ROC-AUC (H), this partial AUC can distinguish between the classifiers (I) but is still constant across different class imbalances, unlike the PR-AUC (G).

area (Figure 4E). If we are only interested in the ER area, we can calculate the AUC over this region: Figure 4F reveals that using this partial AUC allows us to distinguish these classifiers while still retaining the ROC curve's property of consistency across datasets of varying class imbalances (Figure S3).

Precision (PPV) is a linear combination of the coordinates of the ROC curve with FPR weighted by class imbalance

It is not necessarily intuitive to switch between the ROC and PR spaces.[15] Both spaces use TPR, also known as recall, while the differing axes are FPR (ROC) and precision or PPV (PR). Many practitioners find PPV to be more intuitive, as it contains more information as a standalone figure, given that the denominator is the number of predictions. Figure 5 shows how PPV changes throughout the ROC and PR curves for the good ER simulated classifier when the dataset has an imbalance of 1:99 vs. 1:9. The PPV at the same point on the ROC curve for the same simulated classifier can be very different between the two different imbalances, e.g., 0.87 vs. 0.39. This can lead people to believe that the ROC curve is hiding a poor performance on the more imbalanced dataset—in fact, the ROC curve is reflecting an estimate of the classifier performance that is independent of the dataset imbalance in question. As the formula in Figure 5 shows, the PPV is a linear combination of the TPR and FPR where the FPR is increasingly weighted the more imbalanced the dataset is—in other words, the PPV reflects the same points as we would plot in ROC space except that they are represented for a particular imbalance. This appears to be the fundamental misunderstanding when assessing ROC vs. PR spaces for imbalanced datasets: the ROC curve aims to produce an imbalance-independent estimate of the performance of a classifier, while the PR curve is an estimate of performance for a classifier on a specific dataset.

The PR-AUC on a dataset cannot be trivially corrected for class imbalance

We have shown that the PR-AUC for a classifier of a given skill level changes dramatically with class imbalance, while the ROC-AUC is consistent (Figures 3 and 4), and that PPV is calculated using the TPR, FPR, and class imbalance (Figure 5). Given that many people prefer the intuitiveness of PPV, there have been attempts to correct for the class imbalance in the construction of the curve by standardizing both axes[12] or calculating the PR-AUC over a range of different imbalances.[34] Given that these have not been widely adopted, we asked whether we can interpret the PR-AUC of different models at different imbalances by subtracting or normalizing by imbalance, which might be thought to be trivial on intuition.[22]

We show that the PR-AUC changes with class imbalance even when subtracting, normalizing, or scaling the AUC by the random baseline performance (Figure 6). It is not justified to compare classifier performance across datasets with different class imbalances using the PR-AUC by simply subtracting or naively normalizing by the class imbalance. This is a result of how the PR-AUC is calculated, most commonly as a weighted average of precisions, meaning that the extent to which the imbalance affects the average precision will be a function of the shape of the curve. This shows the difficulty in fairly interpreting PR-AUCs across different datasets.

In addition to these AUCs calculated over multiple thresholds, there are other widely used metrics that provide a balance of either precision and recall (e.g., F1 score) or TPR and FPR (G-mean) at a single threshold. The Matthews correlation coefficient (MCC) is

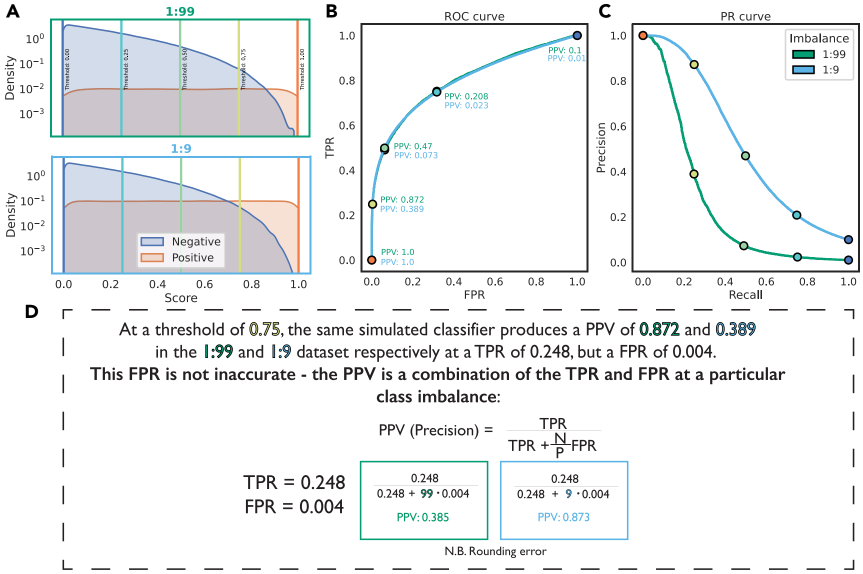

Figure 5. PPV (precision) can be calculated from the coordinates of the ROC curve with the contribution of FPR weighted by the imbalance

(A) Focusing on the good ER classifier shown in Figure 4, the score distributions produced by the same simulation framework for an imbalance of 1:99 and 1:9 are shown normalized to show the different sizes of each subset.

(B and C) The resulting ROC curves are identical (B), while the PR curves are drastically different for the same classifier in different imbalances (C). Many practitioners prefer the precision or PPV axis offered by the PR curve (C).

(D) We annotate the ROC curve with the corresponding PPVs and their position on the PR curve. There is a striking difference in PPV for points with the same TPR and FPR. This is taken as evidence by some that the ROC curve is "hiding" performance differences; however, no information required to calculate the PPV is hidden in the construction of the ROC curve. PPV is simply a combination of these terms at a particular class imbalance, as described by the formula (proof shown in Proof S1).

another single-threshold performance estimate calculated as a correlation coefficient between true and predicted labels. The effect of class imbalance on the estimate of the F1 score, G-mean score, and MCC using a fixed score threshold of 0.5 is shown for each imbalance (Figure S5). For F1 and MCC, the performance estimate and the difference from random (where all instances are predicted positive) change as a function of imbalance, which is expected given their reliance on precision. The G-mean, calculated as the geometric mean of TPR and (1 − FPR), is fixed across differing imbalances.

Case study: Antibody paratope prediction

To contextualize our simulation results, we consider a real imbalanced dataset from an imbalanced domain, antibody paratope prediction. We used a dataset from a paper testing a new paratope prediction model called Paragraph in which the model significantly outperformed a simple baseline of marginal positional frequencies on the basis of the PR-AUC but not the ROC-AUC.[25]

The dataset has a "native" imbalance of 1:2.3, i.e., contains ∼27% positives. In our simulation, we modulated imbalance by sampling randomly from positive and negative score distributions defined as normal distributions. Here, we apply the state-of-the-art (SoTA) paratope prediction method, Paragraph, to produce a score distribution (Figure 7). Akin to our simulation, we over- or undersampled randomly from the negative score distribution, which we refer to as type I negative data enrichment. As in our simulations, we find that random sampling of negative data does not affect the ROC-AUC or ROC-AUC_0.1 (Figures 8A and 8B) but significantly affects the PR-AUC (Figure 8C), as well as its deviation from the random PR-AUC.

In papers examining the effect of class imbalance on species distribution modeling, imbalance is often modulated by enriching for low-scoring instances.[4] This is referred to here as type II negative data enrichment, and it differs in that class imbalance is inextricable from differences in the score distribution—i.e., more imbalanced datasets reflect easier problems because of

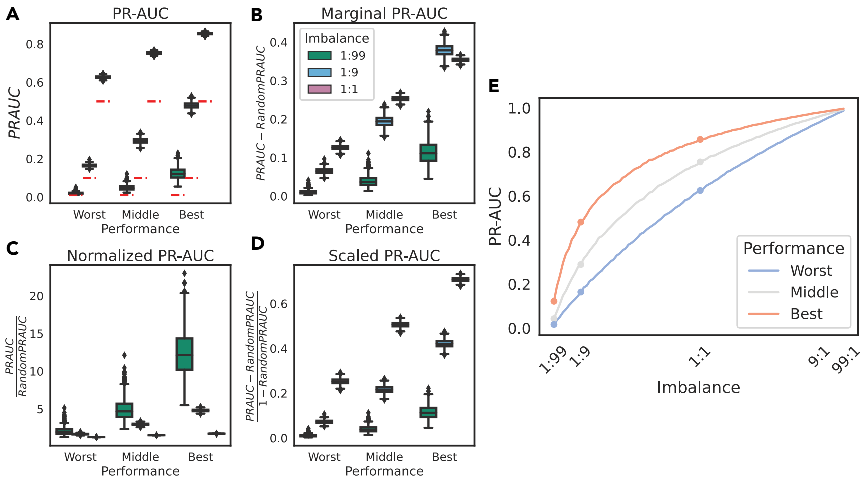

Figure 6. Class imbalance cannot be trivially subtracted out from the PR-AUC, as it affects the calculation of the PR-AUC in a classifier-specific manner

(A) As per Figure 3, PR-AUC changes drastically with class imbalance.

(B–E) We tested three linear transforms of PR-AUC to attempt to account for class imbalance: subtracting the random baseline (class imbalance), marginal PR-AUC (B); dividing PR-AUC by the random baseline, i.e., a fold change, referred to as normalized PR-AUC (C); and min-max scaling of the PR-AUC (D). None can subtract the class imbalance from the AUC. This is because the function of PR-AUC with respect to imbalance is a function of the classifier performance itself (E).

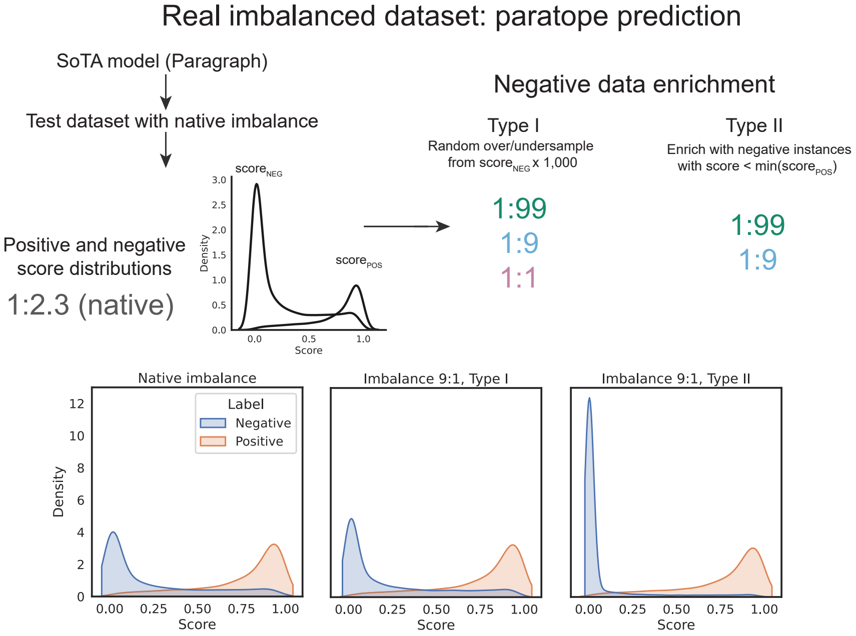

Figure 7. We use a real imbalanced dataset and SoTA model from the domain of antibody paratope prediction to evaluate how imbalance affects performance estimates

We use two types of negative data enrichment: type I negative data enrichment follows our simulation where negative data are drawn randomly from the negative score distribution, whereas type II negative data enrichment changes the imbalance by adding negative instances outside of the positive score distribution.

more trivial negative examples. The study by Cook and Ramadas[35] is another example of a negative data enrichment strategy that conflates class imbalance with score distribution. Type 2 negative data enrichment significantly changes the score distribution such that regardless of classifier skill, the majority of negative instances will have scores below the positive score distribution. Obviously, when a simulated problem is more difficult (i.e., has fewer negative instances outside of the positive score distribution), the ROC-AUC will be lower. Negative data enrichment where new negative instances fall outside of the positive score distribution indeed significantly affects the ROC-AUC, tending toward 1 – imbalance (Figures 8D and 8E): intuitively, this means that the classifier will reach a TPR of 1 at a significantly lower FPR, thus shifting the ROC curve to the left. Contrastingly, the PR-AUC is not affected, as these additional negative instances have scores falling outside of the positive score distribution (Figure 8E).

We further use this example dataset to elucidate the ER area and our selection of an FPR_{max} value. The SoTA model was reported to outperform a simple baseline based on marginal frequencies in the training set with regards to the PR-AUC but not the ROC-AUC. We compare the two (Figures 9A and 9B). In our implementation, the SoTA model does indeed beat the baseline with respective ROC-AUCs of 0.89 and 0.87 but by just 0.02, which is significantly less than the performance difference measured via the PR-AUC, with PR-AUCs of 0.74 and 0.65 (0.09) (Figure 2C).

Our first point is that because of the different properties of PR and ROC spaces, these absolute differences cannot be directly compared. Secondly, we want to emphasize that the small difference between the SoTA and simple models in the ROC-AUC is not attributable to class imbalance, as the same ROC-AUC is achieved when randomly oversampling from the negative class to produce a 1:1 class imbalance (as shown by our type I negative data enrichment in Figures 7 and 8). Thirdly, we want to highlight the region of the ROC curve that corresponds to the top-scoring positive instances, the ER area, in which the SoTA model has a 0.07 advantage over the simple model (Figure 9E). Instances retrieved in this region would be of the most interest to an antibody discovery scientist looking at positions to mutate or not mutate during design. We follow the literature in defining this via an FPR_{max} and have selected an FPR_{max} of 0.1 for the purposes of this work. In practice, this is a value of what an "acceptable" FPR would be in application, beyond which the model behavior is not of interest. In this case, evaluating the models over the ROC_0.1 region tells the scientist how well each model ranks positive and negative instances until 10% of negative instances have been misclassified, which would be the maximum acceptable FPR for their application.

DISCUSSION

Many biological questions require looking for a "needle in the haystack" in that we are attempting to identify rare events in a much larger set of possible choices. It has become an increasingly commonplace opinion that the ROC curve, widely used in diagnostics and signal detection, is inappropriate for such imbalanced datasets in which there is more interest in performance on the minority class. This opinion seems to have its origin in Davis and Goadrich's 2006 paper[15] and contrasts with prior studies identifying the ROC curve as robust to class imbalance.[10] In the present work, we aimed to address this discrepancy.

Our simulations, with both the normal (Figure 3) and beta (Figure 4) distributions, reveal a constant ROC-AUC across differing class imbalances, as is expected where the changing class distribution does not change the underlying score distributions within the class, i.e., the $y \rightarrow x$ domain.[36] When changing the class imbalance will change the score distribution between the classes, this result does not hold. We showed this via our negative data enrichment experiments on a real imbalanced dataset in the domain of antibody paratope prediction (Figure 8): when sampling randomly from the negative score distribution, the average ROC-AUC is not affected by changing the class imbalance, while the PR-AUC and its random baseline change dramatically (Figures 8A–8C). However, if negative data are enriched for by selecting only the lowest score instances that lie outside of the positive score distribution, then the PR-AUC will

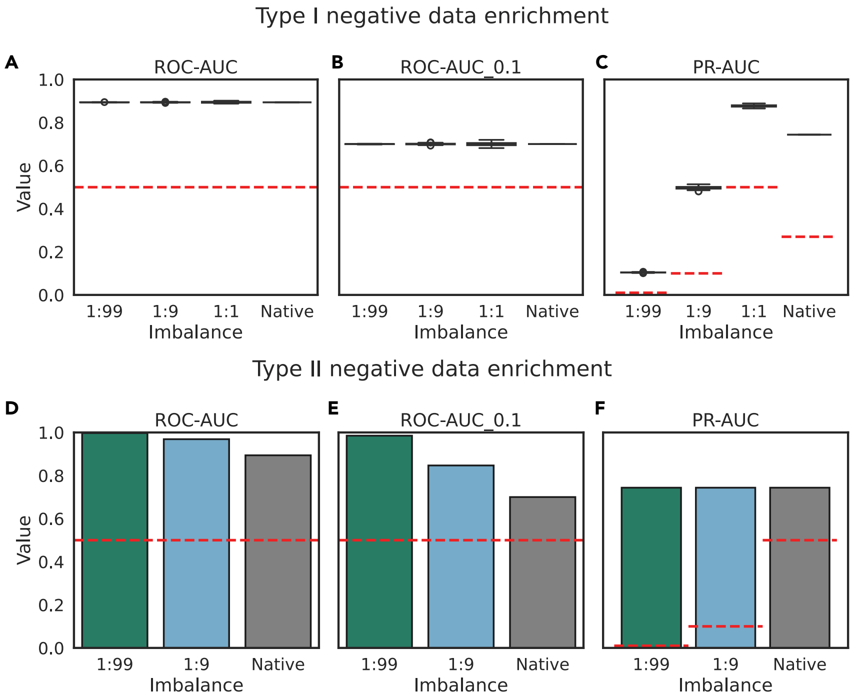

Figure 8. We use a real imbalanced dataset and SoTA model from the domain of antibody paratope prediction to evaluate how imbalance affects performance estimates with two different types of negative data enrichment

(A–C) The first strategy emulates our simulations in that the score distribution does not change (Figure 7): as per our simulations, the ROC-AUC and ROC-AUC_0.1 (averaged across repeats) are not affected by drastically increasing or decreasing imbalance (A and B), while the PR-AUC as well as its random baseline (red dashed line) are (C).

(D–F) In the type II negative data enrichment, the score distribution is changed so that there are many more negative instances outside of the positive score distribution. This results in changes to the ROC-AUC and ROC-AUC_0.1 (D and E) but does not affect the PR-AUC (F).

not be affected, whereas the ROC-AUC will tend toward 1 – the imbalance (Figures 8D–8F). This is expected as, indeed, the score distribution is changing, with the classifier being given a helping hand by the negative data selection strategy. This explains the discrepancy with the species distribution modeling literature, in which the ROC-AUC is found to be inflated by increasing the geographical extent.[4] In such performance evaluations, modulating the imbalance by changing the geographical extent of negatives in the test dataset may inflate the ROC-AUC if the negative data that are added are predominantly low-scoring instances outside of the positive score distribution. Methods like the synthetic minority oversampling technique that sample directly from the feature space should be preferable for changing the class imbalance.[37]

We also see this in the simulations of Cook and Ramadas,[35] where the label and score distributions were sampled from a bivariate normal distribution and the "label" variable was converted to a binary label for ROC curve construction by sampling the upper *n* percentile of label scores according to the class imbalance.[35] When the classes are more imbalanced and *n* is smaller, the score distribution of the positive class is higher than when the class imbalance is lower and *n* is larger. This results in apparent inflation of the ROC-AUC, which is actually just the ROC-AUC correctly reflecting an increase in simulated classifier performance (Figure S4; Table S1).

We interpret our main result, that the ROC curve for a given classifier is unchanged by increasing class imbalance 10-fold in contrast to the PR-AUC, differently to the previous simulation study in the biological sciences domain.[11] The previous authors argue that this is evidence that the ROC-AUC hides information about poor performance: we argue that this is a misunderstanding of the relationship between the ROC and PR curve, as the difference is just a result of the relationship between the TPR and FPR (the ROC curve) and precision or PPV (the PR curve), with the PPV being a reflection of the TPR and FPR

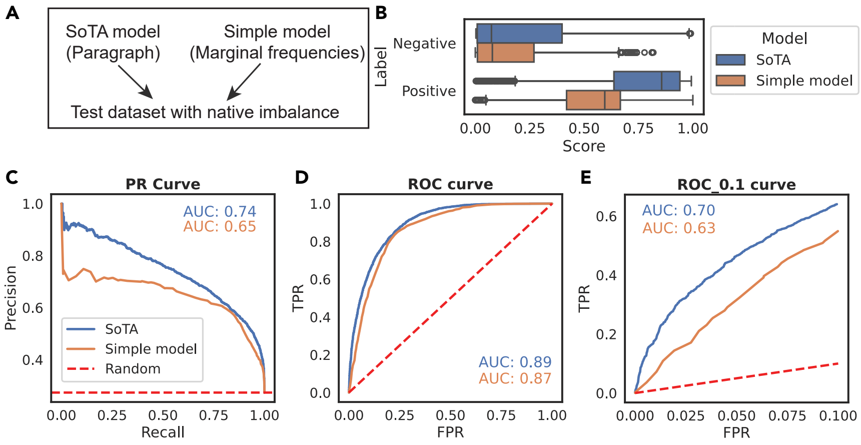

Figure 9. Comparison of a SoTA and simple model for paratope prediction in the PR and ROC spaces

We looked further into how performance estimates differed for a SoTA model and a simple model on the test dataset (A and B). A larger performance difference is observed for the PR-AUC than the ROC-AUC (C) with a difference of 0.09 vs. 0.02 for the ROC curve (D). This performance difference estimate is not attributable to class imbalance. Rather, it reflects both differing properties about the PR-AUC space and the fact that performance is evaluated only where TPR $\leq$ 1. For a performance metric focusing on classifier performance in the score range of the positive distribution, we recommend a partial ROC-AUC evaluated up until a selected FPR_{max}.

(E) The SoTA and simple model show a starker difference in the ER area while retaining the invariance to class imbalance of the full ROC curve.

at a particular class imbalance (Figure 5). Because of the intuitiveness of PPV, the PR-AUC is considered as the ground-truth AUC: the ROC-AUC therefore appears to be inflated with increasing class imbalance, when in reality it is constant against the changing PR-AUC.

In conclusion, we demonstrated that where changing the imbalance does not change the score distribution, ROC-AUC is not changed. Either our simulation results or our interpretations differ from other work in the literature. Where our simulation results differ, this is because of our choice to simulate class imbalance in such a way that it is independent of class imbalance in contrast to, e.g., Cook and Ramadas[35] or Sofaer and colleagues.[4] We argue that our simulation style is the correct simulation style because it allows us to disentangle the effects of class imbalance vs. changes in the difficulty of the problem, which are conflated in these studies. Where score distribution is likely to change with imbalance, the ROC-AUC would change, while the PR-AUC will change if the ranking within the positive score distribution changes.

The realism of this constant score distribution with changing class imbalance depends on application. For example, imagine that a researcher wishes to create a new classifier to diagnose a disease based on a set of features, X. The researcher has a cohort of 100 people with the disease (positives) and wishes to recruit healthy people to act as the negatives. Our simulation reflects the case that the distribution of X among the healthy population will be broadly similar regardless of the size of the cohort, i.e., the underlying distribution of X is the same. If, however, the score distribution changed significantly as the researcher included more negative controls, then the conditions of our simulation would no longer hold; however, we emphasize that in these cases, changes in the ROC-AUC would reflect changes in the score distribution, not an imbalance itself.

Where a researcher is not interested in the performance on the negative class as a whole, we recommend the AUC calculated over the ER area, which is defined via an FPR_{max}.[38] In our test dataset of paratope prediction, this made the differences in performance between a simple and a SoTA model more stark. We imagine another scenario in antibody discovery. The researcher has explored a number of models for predicting the antigen specificity of antibodies they sequenced from a convalescent patient, and they have a fixed budget to express 100 antibodies for further testing from 100,000 antibody sequences available. Thus, they want to minimize the number of FPs within the selected top 100. In this scenario, the researcher should focus on the ER area of the ROC curve when assessing model performance on validation data and could use the ROC-AUC_0.1. Our simulations of classifiers with good or bad ER showed that calculating the AUC up to a small FPR allows you to distinguish between classifiers with different performance over the highest scores (Figure 4). Our selection of an FPR_{max} of 0.1 follows the literature in our field of B cell and T cell epitope prediction.[29,31,33] So that these ER AUCs can be compared across datasets, we would recommend changing this FPR_{max} only by orders of magnitude unless it is extremely well justified based on the proposed application, with the hope that appropriate FPR_{max} values would be adopted for specific tasks.

The partial AUC is already implemented in the widely used ROC-AUC function within the scikit-learn package.[20,38] There are alternatives that apply a transformation to the x axis of the ROC curve such that the ER areas make a disproportionate contribution to the AUC, such as the concentrated ROC (CROC) plot and AUC, which scales the x axis exponentially.[39] However, such methods require selection of the exponent in a data-dependent manner, which raises all of the problems of the PR curve. The "pROC" plot, used in virtual screening applications, logarithmically scales the x axis; however, since the logarithm cannot be evaluated at x = 0, the lower limit of the x axis is N/2 and therefore is not comparable across datasets of differing size (furthermore, it should be noted that pROC is used elsewhere to refer to partial AUCs).[40,41]

Given that the PPV can be calculated from the coordinates of the ROC curve, there is a question of whether we can interpret the resulting PR-AUC given the dataset imbalance. It has been suggested to normalize by the class imbalance or to at least report the PR-AUC alongside the class imbalance to interpret it more fairly. We asked whether we could trivially subtract out the class imbalance by some normalization and found that it is not possible due to a non-linear and classifier-specific relationship between the PR-AUC and class imbalance (Figure 5). This is because the PR-AUC is calculated as the average precision weighted by the change in recall in each interval.[12] Our interpretation is that the contribution of the imbalance to the final AUC will be a function of the changing ratio between the TP and FP, which is classifier specific. Flach and Kull's approach was to calculate a curve with corrected axes, referred to as the PR-gain curve; however, they also have other qualms about the calculation of the AUC itself, which should be addressed separately.[12] The difficulty in extracting class imbalance from the PR-AUC is consistent with the fact that classifiers can be ranked differently according to ROC- and PR-AUCs on the same dataset.[15]

During review, McDermott and colleagues released a preprint in which they relate the PR-AUC and ROC-AUC in a novel way, using their probabilistic forms.[42] They found that the PR-AUC weighs FPs according to the density of datapoints above the score threshold associated with those FPs, i.e., in a classifier-specific way, in contrast to ROC-AUC, which weighs all FPs equally. Their large-language-model-guided literature review also further describes the misunderstanding debunked here, with the Davis and Goadrich paper[15] being the main citation. The authors further clarify that this paper does not make claims about the AUC but rather the ROC and PR curves themselves. Furthermore, they demonstrate that 24% of papers making this claim do not provide citations and that a further 30% cite papers that do not make this claim at all. This work clearly demonstrates that this is a misunderstanding that requires correction.

We hope that our simulations and interpretation clarify the misconception that the ROC-AUC is impacted by imbalanced datasets. In summary, this amounts to a change in the statement that "ROC curves can present an overly optimistic view of an algorithm's performance if there is a large skew in the class distribution," as per Davis and Goadrich,[15] to "ROC curves may look very different from PR curves when there is a large skew in the class distribution due to PR's dependence on this skew." The PR curve is an intuitive and popular visualization tool that is highly informative when comparing different classifiers on the same dataset. Our paper shows that when

Table 2. α and β values used to simulate classifiers with different early retrieval capacities

Early retrieval performance	Positive distribution	Negative distribution
Good	$\alpha = 1, \beta = 1$	$\alpha = 1, \beta = 4$
Bad	$\alpha = 4, \beta = 1$	$\alpha = 1, \beta = 1$

comparing classifiers across datasets with different imbalances, the ROC curve and AUC have been incorrectly characterized as unreliable for imbalanced data when the very opposite is true. It is the PR-AUC that is conditional on the imbalance on the dataset, and there is not a trivial way to disentangle classifier performance from the imbalance in the dataset (though reporting class imbalance alongside the performance estimate is the least that should be done).

EXPERIMENTAL PROCEDURES

Resource availability

Lead contact

Further information and queries should be directed to the lead contact, Bjoern Peters (bpeters@lji.org).

Materials availability

No new materials were generated in this study.

Data and code availability

Code to reproduce the simulations and figures is available at https://github.com/erichardson97/ROC_vs_PR and Zenodo at https://doi.org/10.5281/zenodo.10966607.[43]

Simulation framework

We assume that negative and positive instances are randomly sampled from their respective populations such that sampling is independent of the class imbalance, i.e., the score distribution of either positive or negative instances is not directionally changed by increasing the number of instances of either class. We believe that this is preferable to previous simulation studies that conflate score distributions and imbalance because it allows us to disentangle the two.

We achieve this by drawing positive and negative instances from a normal distribution. We simulate classifiers of differing quality by drawing negative data (N) from a unit-variance, zero-centered normal distribution and positive data (P) from unit-variance normal distributions centered at means of 0.5 (worst classifier), 1 (middle classifier), and 1.5 (best classifier) (Figure 2A). Class imbalance is simulated with a fixed dataset size of 10,000 while varying the proportion of the dataset that is positive (referred to as the imbalance) between one positive to 99 negative instances (1:99) (most imbalanced), one positive instance to 9 negative instances (1:9), and one positive to one negative instance (1:1, a balanced dataset) (Figure 2B). Each simulation is repeated 1,000 times. This is implemented using numpy's random.normal function.

A classifier's ER behavior is its classification performance up to a given FPR, for example up until an FPR of 0.1. To simulate good vs. bad ER, we follow Saito and Rehmsmeier's simulation framework,[11] in which the positive data are simulated as a beta distribution with α and β defined as per Table 2.

Real-world example: Antibody paratope prediction

We used antibody paratope prediction as an example of an imbalanced domain. We used the test dataset from the PECAN paper, as provided by Chinery and colleagues.[25,28] We used the pretrained Paragraph model as distributed by the authors as the SoTA model.[25] We reimplemented the suggested simple model that uses paratope residue frequencies as a comparator (i.e., referred to as marginal frequencies, as they are calculated over the full training set). Paratope residues were calculated using Scipy's KDTree with a distance cutoff of 4.5 Å. For type I negative data enrichment, over- or undersampling was performed randomly from the negative data. For type II negative data enrichment, the lowest-scoring negative instance was repeated in the data to achieve the required imbalance.

ROC-AUC and PR-AUC calculation

The ROC-AUC is calculated using scikit-learn's metrics.*roc_auc_score* function. which uses the trapezoidal method to calculate the AUC. The PR-AUC is calculated using scikit-learn's metrics.*average_precision_score* function. Both functions use the same function to construct the binary classification curve via the calculation of TPR/FPR and TPR/precision at unique thresholds. To calculate the partial ROC-AUC, we use the *max_fpr* argument set to 0.1 within the *roc_auc_score* function, which utilizes the McClish correction to scale the AUC such that random performance is still equal to 0.5.[20,38]

SUPPLEMENTAL INFORMATION

Supplemental information can be found online at https://doi.org/10.1016/j.patter.2024.100994.

ACKNOWLEDGMENTS

Research reported in this publication was supported by the National Cancer Institute of the National Institutes of Health under award no. U24CA248138. The content is solely the responsibility of the authors and does not necessarily represent the official views of the National Institutes of Health. This project has been funded in whole or in part with federal funds from the National Institute of Allergy and Infectious Diseases, National Institutes of Health, Department of Health and Human Services under contract no. 75N93019C00001.

AUTHOR CONTRIBUTIONS

E.R. and B.P. devised the simulations. E.R. wrote the code. All authors contributed to the writing of the paper.

DECLARATION OF INTERESTS

The authors declare no competing interests.

Received: December 1, 2023
Revised: March 5, 2024
Accepted: May 3, 2024
Published: May 31, 2024

REFERENCES

1. Gainza, P., Sverrisson, F., Monti, F., Rodolà, E., Boscaini, D., Bronstein, M.M., and Correia, B.E. (2020). Deciphering interaction fingerprints from protein molecular surfaces using geometric deep learning. Nat. Methods *17*, 184–192. https://doi.org/10.1038/s41592-019-0666-6.
2. Williams, N.P., Rodrigues, C.H.M., Truong, J., Ascher, D.B., and Holien, J.K. (2023). DockNet: high-throughput protein–protein interface contact prediction. Bioinformatics *39*, btac797. https://doi.org/10.1093/bioinformatics/btac797.
3. Carter, H., Chen, S., Isik, L., Tyekucheva, S., Velculescu, V.E., Kinzler, K.W., Vogelstein, B., and Karchin, R. (2009). Cancer-Specific High-Throughput Annotation of Somatic Mutations: Computational Prediction of Driver Missense Mutations. Cancer Res. *69*, 6660–6667. https://doi.org/10.1158/0008-5472.CAN-09-1133.
4. Sofaer, H.R., Hoeting, J.A., and Jarnevich, C.S. (2019). The area under the precision-recall curve as a performance metric for rare binary events. Methods Ecol. Evol. *10*, 565–577. https://doi.org/10.1111/2041-210X.13140.
5. Thölke, P., Mantilla-Ramos, Y.-J., Abdelhedi, H., Maschke, C., Dehgan, A., Harel, Y., Kemtur, A., Mekki Berrada, L., Sahraoui, M., Young, T., et al. (2023). Class imbalance should not throw you off balance: Choosing the right classifiers and performance metrics for brain decoding with imbalanced data. Neuroimage *277*, 120253. https://doi.org/10.1016/j.neuroimage.2023.120253.
6. Daberdaku, S., and Ferrari, C. (2019). Antibody interface prediction with 3D Zernike descriptors and SVM. Bioinformatics *35*, 1870–1876. https://doi.org/10.1093/bioinformatics/bty918.

CellPress
OPEN ACCESS

7. Meysman, P., Barton, J., Bravi, B., Cohen-Lavi, L., Karnaukhov, V., Lilleskov, E., Montemurro, A., Nielsen, M., Mora, T., Pereira, P., et al. (2023). Benchmarking solutions to the T-cell receptor epitope prediction problem: IMMREP22 workshop report. ImmunoInformatics *9*, 100024. https://doi.org/10.1016/j.immuno.2023.100024.
8. He, H., and Garcia, E.A. (2009). Learning from Imbalanced Data. IEEE Trans. Knowl. Data Eng. *21*, 1263–1284. https://doi.org/10.1109/TKDE.2008.239.
9. Provost, F., and Fawcett, T. (2001). Robust Classification for Imprecise Environments. Mach. Learn. *42*, 203–231. https://doi.org/10.1023/A:1007601015854.
10. Swets, J.A. (1988). Measuring the Accuracy of Diagnostic Systems. Science *240*, 1285–1293. https://doi.org/10.1126/science.3287615.
11. Saito, T., and Rehmsmeier, M. (2015). The Precision-Recall Plot Is More Informative than the ROC Plot When Evaluating Binary Classifiers on Imbalanced Datasets. PLoS One *10*, e0118432. https://doi.org/10.1371/journal.pone.0118432.
12. Flach, P., and Kull, M. (2015). Precision-Recall-Gain Curves: PR Analysis Done Right. In Advances in Neural Information Processing Systems 28 (NIPS 2015) (Curran Associates, Inc.).
13. Hernández-Orallo, J., Flach, P., and Ferri, C. (2012). A Unified View of Performance Metrics: Translating Threshold Choice into Expected Classification Loss. J. Mach. Learn. Res. *13*, 2813–2869.
14. Mason, S.J., and Graham, N.E. (2002). Areas beneath the relative operating characteristics (ROC) and relative operating levels (ROL) curves: Statistical significance and interpretation. Q. J. R. Meteorol. Soc. *128*, 2145–2166. https://doi.org/10.1256/003590002320603584.
15. Davis, J., and Goadrich, M. (2006). The relationship between Precision-Recall and ROC curves. In Proceedings of the 23rd international conference on Machine learning - ICML '06 (ACM Press), pp. 233–240. https://doi.org/10.1145/1143844.1143874.
16. Branco, P., Torgo, L., and Ribeiro, R.P. (2016). A Survey of Predictive Modeling on Imbalanced Domains. ACM Comput. Surv. *49*, 1–50. https://doi.org/10.1145/2907070.
17. Brownlee, J. (2020). Imbalanced Classification with Python: Better Metrics, Balance Skewed Classes, Cost-Sensitive Learning (Machine Learning Mastery).
18. He, H., and Ma, Y. (2013). Imbalanced Learning: Foundations, Algorithms, and Applications (John Wiley & Sons).
19. López, V., Fernández, A., García, S., Palade, V., and Herrera, F. (2013). An insight into classification with imbalanced data: Empirical results and current trends on using data intrinsic characteristics. Inf. Sci. *250*, 113–141. https://doi.org/10.1016/j.ins.2013.07.007.
20. Pedregosa, F., Varoquaux, G., Gramfort, A., Michel, V., Thirion, B., Grisel, O., Blondel, M., Prettenhofer, P., Weiss, R., Dubourg, V., et al. (2011). Scikit-learn: Machine Learning in Python. J. Mach. Learn. Res. *12*, 2825–2830.
21. Chicco, D., and Jurman, G. (2020). The advantages of the Matthews correlation coefficient (MCC) over F1 score and accuracy in binary classification evaluation. BMC Genom. *21*, 6. https://doi.org/10.1186/s12864-019-6413-7.
22. Jeni, L.A., Cohn, J.F., and De La Torre, F. (2013). Facing Imbalanced Data–Recommendations for the Use of Performance Metrics. In 2013 Humaine Association Conference on Affective Computing and Intelligent Interaction, pp. 245–251. https://doi.org/10.1109/ACII.2013.47.
23. Ozenne, B., Subtil, F., and Maucort-Boulch, D. (2015). The precision–recall curve overcame the optimism of the receiver operating characteristic curve in rare diseases. J. Clin. Epidemiol. *68*, 855–859. https://doi.org/10.1016/j.jclinepi.2015.02.010.
24. Poisot, T. (2023). Guidelines for the prediction of species interactions through binary classification. Methods Ecol. Evol. *14*, 1333–1345. https://doi.org/10.1111/2041-210X.14071.
25. Chinery, L., Wahome, N., Moal, I., and Deane, C.M. (2023). Paragraph—antibody paratope prediction using graph neural networks with minimal feature vectors. Bioinformatics *39*, btac732. https://doi.org/10.1093/bioinformatics/btac732.
26. Dai, B., and Bailey-Kellogg, C. (2021). Protein interaction interface region prediction by geometric deep learning. Bioinformatics *37*, 2580–2588. https://doi.org/10.1093/bioinformatics/btab154.
27. Del Vecchio, A., Deac, A., Liò, P., and Veličković, P. (2021). Neural message passing for joint paratope-epitope prediction. Preprint at arXiv. https://doi.org/10.48550/arXiv.2106.00757.
28. Pittala, S., and Bailey-Kellogg, C. (2020). Learning context-aware structural representations to predict antigen and antibody binding interfaces. Bioinformatics *36*, 3996–4003. https://doi.org/10.1093/bioinformatics/btaa263.
29. Stranzl, T., Larsen, M.V., Lundegaard, C., and Nielsen, M. (2010). NetCTLpan: pan-specific MHC class I pathway epitope predictions. Immunogenetics *62*, 357–368. https://doi.org/10.1007/s00251-010-0441-4.
30. Fawcett, T. (2004). ROC graphs: Notes and practical considerations for researchers. Mach. Learn. *31*, 1–38.
31. Clifford, J.N., Høie, M.H., Deleuran, S., Peters, B., Nielsen, M., and Marcatili, P. (2022). BepiPred-3.0: Improved B-cell epitope prediction using protein language models. Protein Sci. *31*, e4497. https://doi.org/10.1002/pro.4497.
32. Li, Y., Sackett, P.W., Nielsen, M., and Barra, C. (2023). NetAllergen, a random forest model integrating MHC-II presentation propensity for improved allergenicity prediction. Preprint at bioRxiv. https://doi.org/10.1101/2022.09.22.509069.
33. Nilsson, J.B., Kaabinejadian, S., Yari, H., Peters, B., Barra, C., Gragert, L., Hildebrand, W., and Nielsen, M. (2023). Machine learning reveals limited contribution of trans-only encoded variants to the HLA-DQ immunopeptidome. Commun. Biol. *6*, 442. https://doi.org/10.1038/s42003-023-04749-7.
34. Bradley, A.P., Duin, R.P.W., Paclik, P., and Landgrebe, T.C.W. (2006). Precision-recall operating characteristic (P-ROC) curves in imprecise environments. In 18th International Conference on Pattern Recognition (ICPR'06), pp. 123–127. https://doi.org/10.1109/ICPR.2006.941.
35. Cook, J., and Ramadas, V. (2020). When to consult precision-recall curves. The Stata Journal *20*, 131–148. https://doi.org/10.1177/1536867X20909693.
36. Fawcett, T., and Flach, P.A. (2005). A Response to Webb and Ting's On the Application of ROC Analysis to Predict Classification Performance Under Varying Class Distributions. Mach. Learn. *58*, 33–38. https://doi.org/10.1007/s10994-005-5256-4.
37. Chawla, N.V., Bowyer, K.W., Hall, L.O., and Kegelmeyer, W.P. (2002). SMOTE: Synthetic Minority Over-sampling Technique. J. Artif. Intell. Res. *16*, 321–357. https://doi.org/10.1613/jair.953.
38. McClish, D.K. (1989). Analyzing a Portion of the ROC Curve. Med. Decis. Making *9*, 190–195. https://doi.org/10.1177/0272989X8900900307.
39. Swamidass, S.J., Azencott, C.-A., Daily, K., and Baldi, P. (2010). A CROC stronger than ROC: measuring, visualizing and optimizing early retrieval. Bioinformatics *26*, 1348–1356. https://doi.org/10.1093/bioinformatics/btq140.
40. Clark, R.D., and Webster-Clark, D.J. (2008). Managing bias in ROC curves. J. Comput. Aided Mol. Des. *22*, 141–146. https://doi.org/10.1007/s10822-008-9181-z.
41. Robin, X., Turck, N., Hainard, A., Tiberti, N., Lisacek, F., Sanchez, J.-C., and Müller, M. (2011). pROC: an open-source package for R and S+ to analyze and compare ROC curves. BMC Bioinf. *12*, 77. https://doi.org/10.1186/1471-2105-12-77.
42. McDermott, M.B.A., Hansen, L.H., Zhang, H., Angelotti, G., and Gallifant, J. (2024). A Closer Look at AUROC and AUPRC under Class Imbalance. Preprint at arXiv. https://doi.org/10.48550/arXiv.2401.06091.
43. Richardson, E. (2024). Notebook Accompanying "The ROC-AUC Accurately Assesses Imbalanced datasets." (Zenodo). https://doi.org/10.5281/zenodo.10966607.

Patterns, Volume *5*

Supplemental information

The receiver operating characteristic curve accurately assesses imbalanced datasets

Eve Richardson, Raphael Trevizani, Jason A. Greenbaum, Hannah Carter, Morten Nielsen, and Bjoern Peters

Supplemental Experimental Procedures

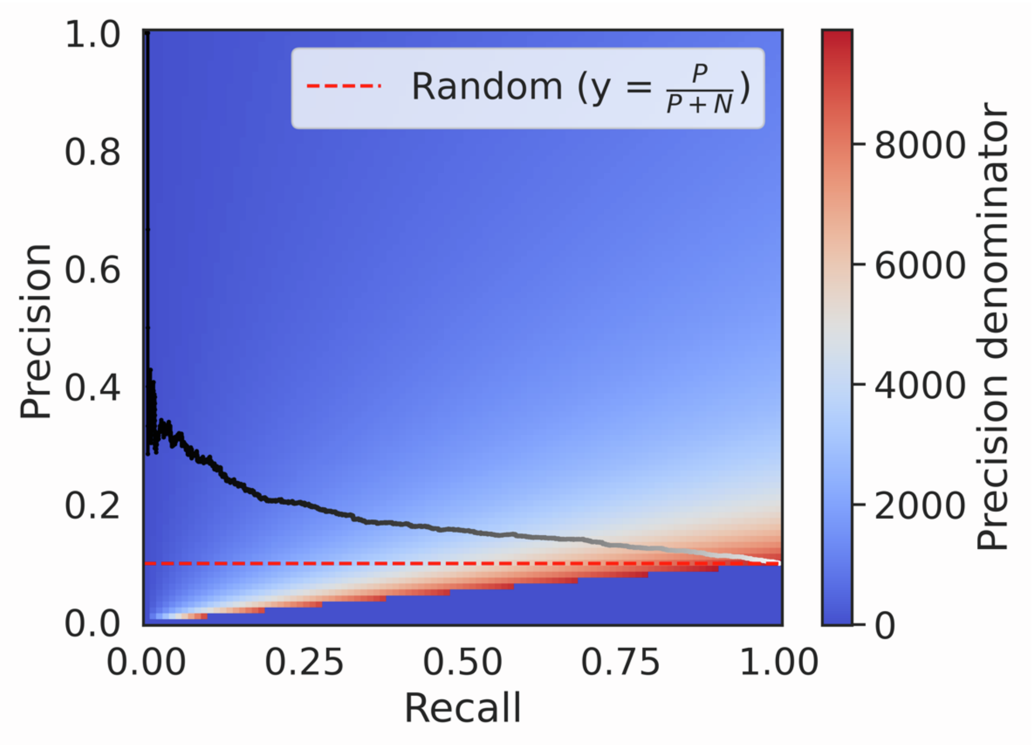

Figure S1: PR space is non-linear, as the denominator of Precision ($\hat{P}$) changes while moving through PR space which means that Precision does not vary uniformly with TPR. At the lowest score threshold, $\hat{P}$ will equal P+N (i.e. everything predicted as positive), and will decrease from right to left across the plot (until nothing is predicted as positive). The dark area below the random baseline corresponds to the unachievable area for a given TPR level, defined by the imbalance in the dataset [1]. With an imbalance of 0.1, Precision is only defined at TPR levels greater than 0.053 according to the formula presented in Boyd et al (2012). This limits the extent to which classifiers can be worse than random.

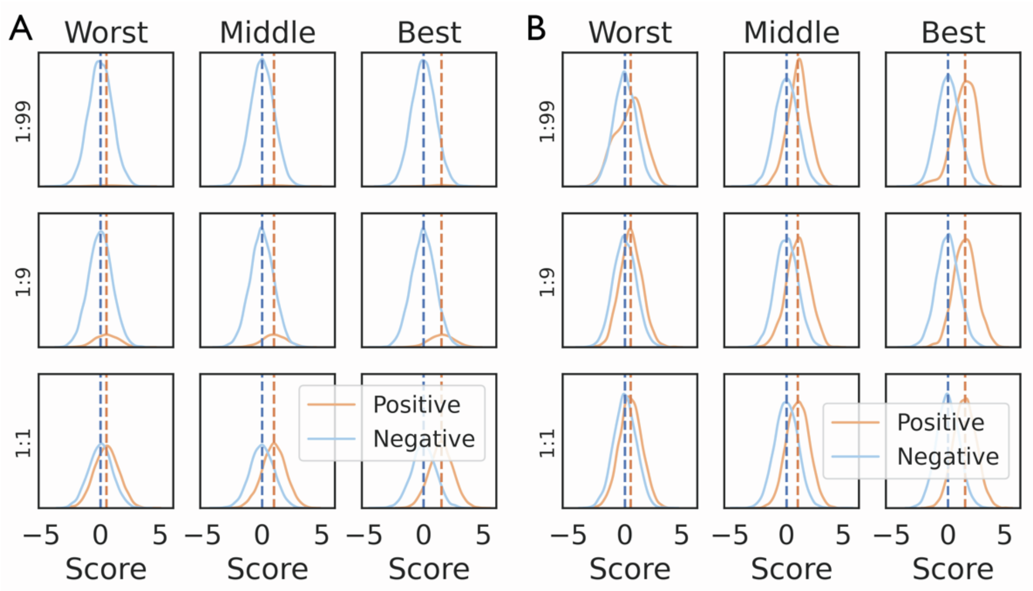

Figure S2: total score distributions across all simulations for each class imbalance (rows) and each classifier (columns), normalized across the entire dataset (A) and on a per-class basis (B). The underlying score distributions for the positive and negative class is not changed by changing class imbalance.

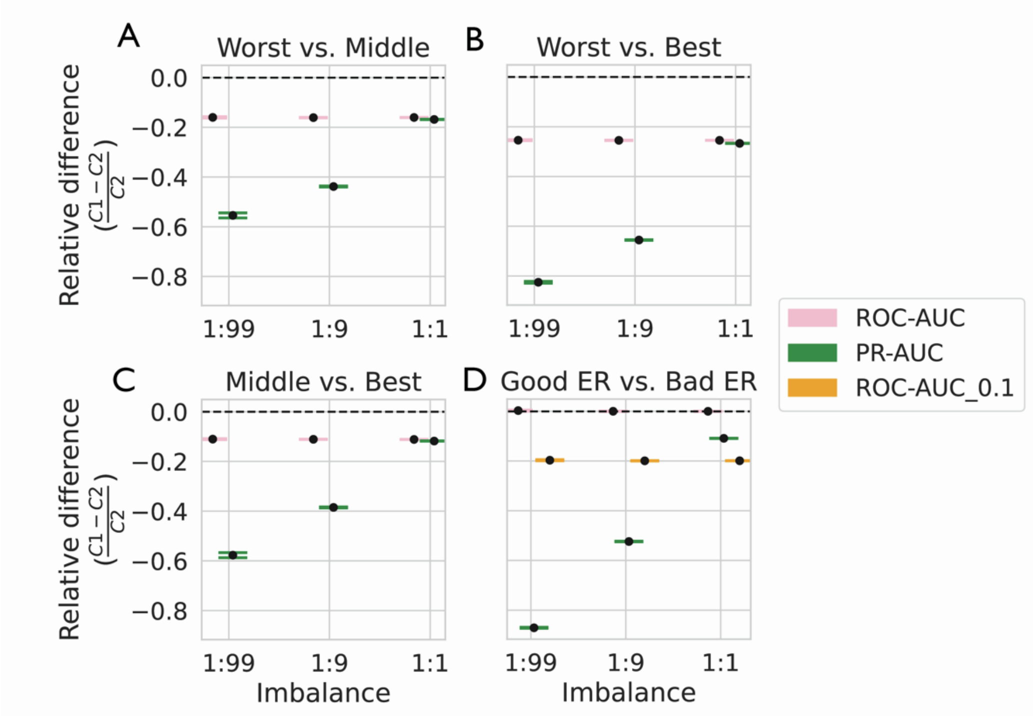

Figure S3: classifier performance differences between each pair of classifiers, "Worst", "Middle", "Best" (A, B, C) and the Good vs. Bad ER (D). The metric for the better classifier (C2) is subtracted from the worse classifier (C1) and divided by the performance estimate of the better classifier to calculate the relative difference, (C1 -

C2)/C2, for each imbalance. ROC-AUC's estimate of the difference in performance is constant as expected given that the absolute performance estimation is constant. Meanwhile, the difference in the performance per the PR-AUC differs by as much as 80% of the value of the better classifier. While the ROC-AUC (pink) is not able to distinguish between classifiers with good and bad early retrieval and symmetrical distributions because the corresponding curves cross (D), a partial AUC over the early retrieval region such as ROC-AUC_0.1 (orange), allows us to distinguish between the two (i.e. non-zero difference), irrespective of the underlying class distribution. The PR-AUC (green) is sensitive to early retrieval behavior due to the fact that it explicitly only considers the positive class, however it is also highly sensitive to the underlying class imbalance.

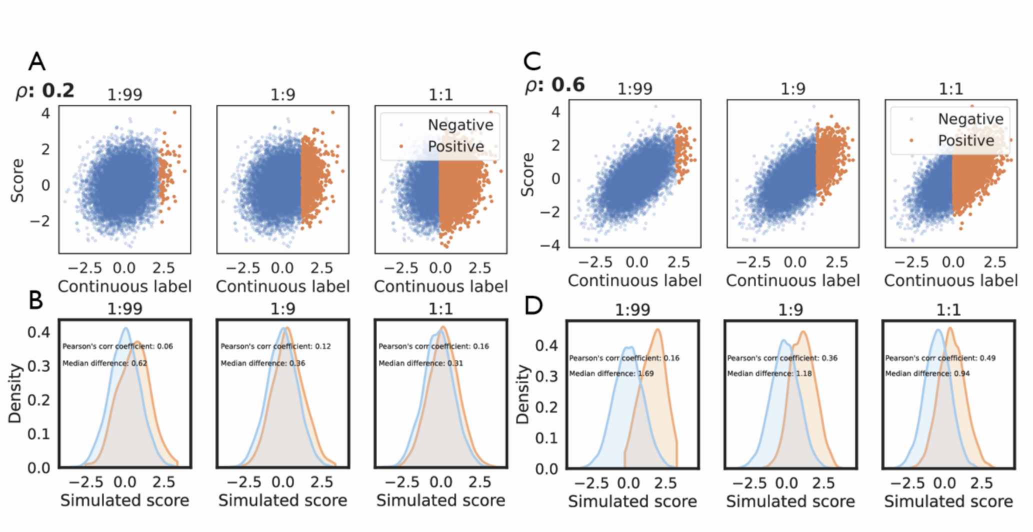

Figure S4: we recreated the simulation framework from Cook & Ramadas (2020), in which classifier performance is simulated via the covariance between two zero-centred Normal distributions (implemented with numpy's multivariate_normal function)[2]. Shown here are a covariance of 0.2 (A and B) and 0.6 (C and D). In this simulation, we convert the continuous label into a binary label via selecting a threshold above which the top 1%, 10% or 50% of instances are labelled as positive (A, C). This results in an improvement in the actual simulated classifier performance with increasing imbalance where the correlation coefficient between the two distributions is non-zero, as then the top 1%, 10% or 50% of instances by the label distribution is also likely to be among the top scoring instances in the score distribution (B and D).

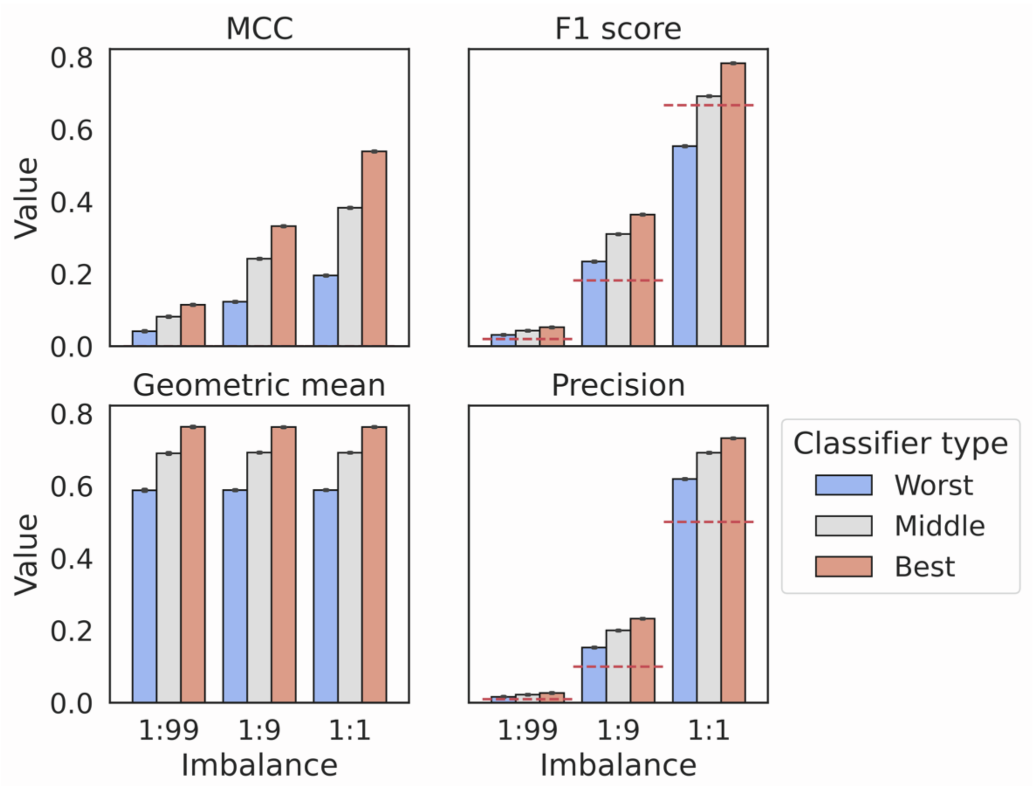

Figure S5: we calculated single-threshold performance estimates for our simulated classifiers, using an arbitrary score threshold of 0.5 for each classifier. Red dashed lines indicate the random baseline where all instances are predicted as positive, i.e. where Precision = Imbalance and Recall = 1. For MCC and Geometric Mean, the random baseline is 0. For the PR metrics (MCC and F1-score), classifier performance and deviation from random baseline is a function of imbalance. The geometric mean is constant across different imbalances (geometric mean of TPR and (1-FPR)).

	Imbalance		
Correlation	1:99	1:9	1:1
0.0	0.50 / 0.50	0.50 / 0.50	0.50 / 0.50
0.2	0.66 / 0.65	0.61 / 0.61	0.59 / 0.59
0.4	0.79 / 0.79	0.72 / 0.72	0.68 / 0.68
0.6	0.90 / 0.90	0.82 / 0.82	0.78 / 0.78
0.8	0.97 / 0.97	0.92 / 0.92	0.88 / 0.88

Table S1: median ROC-AUC across our 1,000 simulations vs. reported ROC-AUC by Cook & Ramadas (2020), with varying classifier performance (correlation between the two distributions which Cook & Ramadas denote as ρ) and imbalance. Note that the

ROC-AUC looks to be “inflated” with increasing class imbalance: this is just the ROC-AUC correctly reflecting the fact that the simulated classifier performance is indeed better in more imbalanced datasets in this simulation framework, due to the imbalance changing the score distribution. This effect is more pronounced where the two distributions are more correlated, e.g., the difference from a balanced to the least balanced simulation is 0.7 with a covariance of 0.2, and 0.12 with a covariance of 0.8.

Supplementary Proof 1:

$$TPR = \frac{TP}{P}$$

$$FPR = \frac{FP}{N}$$

$$PPV = \frac{TP}{TP + FP}$$

$$PPV = \frac{P \cdot TPR}{P \cdot TPR + N \cdot FPR}$$

$$PPV = \frac{TPR}{TPR + \frac{N}{P} \cdot FPR}$$

1. Boyd, K., Santos Costa, V., Davis, J., and Page, C.D. (2012). Unachievable Region in Precision-Recall Space and Its Effect on Empirical Evaluation. Proc. Int. Conf. Mach. Learn. Int. Conf. Mach. Learn. *2012*, 349.

2. Cook, J., and Ramadas, V. (2020). When to consult precision-recall curves. Stata J. *20*, 131–148. 10.1177/1536867X20909693.
